# Cardiac Monitoring: Then and Now

**DOI:** 10.19102/icrm.2019.100909

**Published:** 2019-09-15

**Authors:** Rahul N. Doshi

**Affiliations:** ^1^Division of Cardiology, Keck School of Medicine, University of Southern California, Los Angeles, CA, USA

**Keywords:** Cardiac monitoring, consumer, implantable cardioverter-defibrillator, remote monitoring

In this special edition of *The Journal of Innovations in Cardiac Rhythm Management*, we focus on the increasing applications of remote monitoring in the world of electrophysiology. Ambulatory monitoring has long been a mainstay in the diagnosis and treatment of arrhythmias. The Holter monitor was developed by experimental physicists Norman J. Holter and Bill Glasscock in the 1950s and commercially released in 1962.^1^ However, today’s ambulatory monitors bear little resemblance to the original design, with current ambulatory patch monitoring ubiquitous.^2^ Similarly, cardiac implantable electronic devices were only able to be evaluated during in-person office visits until remote monitoring capabilities were made available, and the option for remote monitoring is now included in almost every implanted device.^3^ Overall, cardiac monitors are becoming smaller, more accurate, and more sophisticated.^4^ The power of such technologies for the evaluation and validation of our treatments is no more evident than when one thinks of the sheer number of patients evaluated—the landmark pivotal studies that formed important platforms in the approval process for devices such as the implantable defibrillator enrolled only hundreds or thousands of patients, while hundreds *of* thousands of patients are involved trials such as the ALTITUDE study.^5^ Remote monitoring has greatly increased the ability to diagnose ambulatory arrhythmias and manage cardiac implantable electronic devices alike—both of which are fundamental parts of the duties of practicing cardiac rhythm specialists.

Despite these amazing advancements, however, this may only represent the “tip of the iceberg” given that we continue to move forward toward incorporating patient-centric and consumer-driven technologies in the world of arrhythmia management.^6–8^ Smartphone electrocardiogram devices and apps that link the patient with their implanted device are being increasingly used and validated. In particular, the Apple Heart Study rapidly enrolled approximately 450,000 patients to evaluate the accuracy of the Apple Watch (Apple Inc., Cupertino, CA, USA) for detecting atrial fibrillation.^8^

The articles featured in this issue span this increasingly diverse area within electrophysiology. Amuthan et al.^9^ present a fascinating evaluation of patch monitoring in the hospital setting, which has the potential to improve detection as well as make every hospital bed into a “telemetry” bed. Ferrara et al. offer a unique case study involving His-bundle pacing and device follow-up with remote monitoring.^10^ Our group working with a platform that considered both an implantable cardioverter-defibrillator and implantable pulmonary artery pressure sensor (MERLIN.NET™ and CardioMems™; Abbott Laboratories, Chicago, IL, USA) used this setup to demonstrate a downward-trending relationship between pulmonary artery pressure and device therapy for ventricular arrhythmias.^11^ Finally, Doshi et al. demonstrate in a case report the accuracy of an Apple Watch Series 4 (Apple Inc., Cupertino, CA, USA) in detecting atrial fibrillation confirmed with implantable cardioverter-defibrillator interrogation.^12^

I sincerely hope that you enjoy the collection of manuscripts presented in this issue of the journal and hope also that they trigger additional ideas for patient care and future investigation. Cheers!
